# Magnitude of early postoperative hypoxemia and its associated factors among adult patients who undergo emergency surgery under general anesthesia at Jimma Medical Center, Jimma, Southwest Ethiopia, 2021: a prospective observational study

**DOI:** 10.1186/s13741-022-00288-7

**Published:** 2023-01-03

**Authors:** Mitiku Berhanu, Negashu Dadi, Berhanu Mengistu, Zemenu Muluken, Ashenafi Tolesa, Tajera Tageza, Megersa Kalbesa, Gezahegn Tesfaye, Belay Zawdie

**Affiliations:** 1 Department of Anesthesia, Institute of Health Science, Jimma University, Jimma, Ethiopia; 2 Department of Anesthesia, College of Health Science, Hawassa University, Hawassa, Ethiopia; 3 Department of Biomedical Science, Institute of Health Science, Jimma University, Jimma, Ethiopia

**Keywords:** EPH, Postoperative recovery period, Circadian rhythm of lung, Jimma Medical Center

## Abstract

**Purpose:**

Emergency surgical procedures involve considerable risks. Among these, early postoperative hypoxemia (EPH) is a frequent anesthetic complication in the post-anesthetic care unit (PACU). There is a great concern for EPH among health professionals, specifically, those providing emergency surgery during the nighttime. This raised anesthesia-ended time-related risk of EPH question. Thus, this study aimed to determine the magnitude of EPH and its associated factors among adult patients who undergo emergency surgery under general anesthesia.

**Methods:**

A prospective observational study through a consecutive sampling technique was conducted. Binary logistic regression analysis was used to identify associated risk factors. All variables that were found statistically significant on bivariable analysis were entered into a multivariable logistic regression analysis.

**Result:**

Of 352 patients who had undergone emergency surgery, 149 (42.3%) patients developed EPH. Factors significantly associated with EPH were anesthesia ended during nighttime (AOR = 1.76, 95%CI [1.01, 3.05], *p* = 0.045), ASA III (AOR = 12.35, 95%CI: [4.5, 34.02], *p* ≤ 0.001), age greater than 55 (AOR = 3.2, 95%CI: [1.7, 5.91], *p* ≤ 0.001), surgery duration greater than 2 h (AOR = 2.012, 95%CI: [1.2, 3.51], *p* = 0.014), hypotension (AOR = 10.3, 95%CI: [2.4, 44.16], *p* = 0.002), muscular strength score zero (AOR = 2.944, 95%CI: [1.8, 4.82], *p* ≤ 0.001), and preoperative oxygen saturation less than 95% (AOR = 2.371, 95%CI: [1.35,4.16], *p* = 0.003).

**Conclusion:**

The magnitude of EPH among patients who have undergone emergency surgery was high and thus recommended that oxygen should be provided timely to decrease it. Identified risk factors were night-time ended anesthesia, ASA III, age greater than 55, surgery duration greater than 2 h, hypotension, muscular strength score zero, and preoperative oxygen saturation less than 95%. This study found anesthesia ended during early morning favors early morning early postoperative hypoxemia (EMEPH). To avert EMEPH, the anesthetist should avoid factors that favor the circadian rhythm of the lung-based early morning anesthesia augmented EPH.

## Introduction

Nowadays surgery under anesthesia, outcomes are becoming increasingly fine due to advancements in diagnosing, treating, and preventing the adverse events that accompany anesthesia and surgery. However, surgical procedures still involve considerable risks (Faraj et al. 2012; Alexandra et al. 2015). Among those, postoperative hypoxemia is the frequent anesthetic complication in the post-anesthetic care unit (PACU), with incidences ranging from 14 to 80% (Canet et al. 1989; Moller and Wittrup 1990; Wang et al. 2013).

Early postoperative hypoxemia (EPH) is defined as insufficient oxygen in the blood (Pearl 1992). It can be appreciated by low arterial partial pressure of oxygen (PaO2) level in the arterial blood less than 60 mmHg or with the aid of pulse oximetry (SpO2) below 90% (Marcondes et al. 2006; Smith et al. 2016; Filho et al. 2001). Many perioperative factors may induce the development and progression of hypoxemia, such as preoperative underlying diseases, surgical injury, anesthetics, and postoperative respiratory events (Tang et al. 2019). Different studies have reported that the incidence of hypoxemia in patients who have undergone upper abdominal surgery is higher and upper abdominal surgery is known to be associated with a high incidence of postoperative pulmonary complications (Marcondes et al. 2006; Meiklejohn et al. 1987). Serious consequences, such as arrhythmias and abnormal blood pressure may follow hypoxemia (Wheatley 1990). Severe hypoxemia has a potential to kill or leave the patient with devastating neurological handicaps, can cause neurological damage, and increases unplanned intensive care unit (ICU) admissions, morbidity, mortality, and hospital length of stay (Maity et al. 2010; Uakritdathikarn and Chongsuvivatwong 2008; Rose et al. 1994; Lawrence et al. 1995).

Indeed, there are different studies done pertaining to the incidence and associated factors of EPH; there are factors that are not studied at all with hypoxemia yet. The particular factor this study gave a due focus on was the time of the day anesthesia ended. Some clinical studies suggested that handling the patients during the nighttime is linked to the worse outcome, favoring the occurrence of complications (Peberdy et al. 2008 Feb; Eskesen et al. 2018). However, these studies did not scrutinize specifically the incidence of EPH after nighttime emergency surgery, which was given due emphasis in this study.

Different studies found different factors account for EPH, broadly sorted as anesthesia, surgery, and patient-related factors (Filho et al. 2001; Quintero-cifuentes and Pérez-lópez 2018a). However, there is still a great concern for EPH among health professionals; specifically, those providing emergency health care for patients who undergo emergency surgery, even when there are no previously found significant factors. This is vividly evidenced when carrying out emergency surgery during the nighttime, and this raised time anesthesia ended-related EPH question. Therefore, the robust motive that made us undertake this study was our high curiosity on the association of anesthesia ended during the nighttime with EPH; which was not ever studied. So this study was aimed at generating sufficient knowledge concerning this aspect of hypoxemia after emergency surgery.

Moreover, to the best of our knowledge, we can say there was nobody of paper that studied EPH among patients who have undergone emergency surgery only under general anesthesia (GA) with endo-tracheal tube (ETT) exclusively.

## Methods

Institution-based, prospective observational study design was conducted at Jimma Medical Center (JMC), a tertiary teaching medical center located in Jimma town, Southwest of Ethiopia. The study was conducted from March 01 to May 30, 2021. All consecutive patients who were undergoing emergency operation under GA with ETT during the study period were included in the study. Patients who underwent emergency surgery under GA and not admitted to PACU, on oxygen therapy before surgery, or sustained injury around the chest that could lead to hypoxemia (e.g., patients with pneumothorax) and patients who cannot communicate were excluded from the study after being verified by checking from their medical records and observations.

Independent factors included in the body of structured questionnaire were reached by reviewing the patient’s charts and interviewing the patients. Anesthesia and surgery-related data (e.g., type of volatile anesthetics, time of the day anesthesia administered, intravenous (IV) anesthetics, intraoperative analgesia, duration and type of surgery, and surgeon experience) was obtained from the anesthetic record sheet, patient’s chart, and operation registration books. Patients were interviewed by the data collectors to self-report socio-demographic factors and patient-related factors of EPH. During the interview of the patients, the questionnaire prepared in English was translated into Afan Oromo and Amharic, which are spoken by local community. Like other independent factors, the time of the day anesthesia ended was collected from the anesthesia chart too.

A manual pulse oximetry was used to record SpO2 during transferring the patient from the operation room (OR) to PACU and in-built pulse oximetry machine monitor was used to measure the dependent variable at PACU. Peripheral arterial oxygen saturation, heart rate (HR), blood pressure, and body temperature were also recorded with the advent of the patient monitor (except body temperature, which was recorded manually using a thermometer) at PACU at admission and at 5-min intervals for 20 min.

At the same time, patient’s sedation score (0 = agitated, 1 = calm, alert, 2 = sleepy and awaking after verbal command, 3 = sleepy and awaking after tactile stimulation, and 4 = comatose), ventilation score (0 = apnea, 1 = limited breathing, respiratory obstruction, bronchospasm or HR below 8 breath per minute, and 2 = free breathing), muscular strength score (1 = keeps grip for > 15 s or 0 = does not keep grip or keeps it for less than 15 s), and verbal pain score (0 = no pain or no answer, 1 = mild pain, 2 = moderate pain and 3 = severe pain) were recorded (Filho et al. 2001).

Covid-19 infection prevention was implemented by all bodies (principal investigator, supervisor, data collectors, and participants) involved in this study. This was achieved by using face mask and sanitizer after touching the patient’s charts and other materials and practicing recommended physical distance. For data collectors, COVID-19 infection prevention was part of the training, as well as they were enforced to wear face mask during contact with participants to collect data. Appropriate hand hygiene before touching a patient and after touching a patient and patient’s surrounding was implemented by data collectors using water and soap.

Two BSc anesthetists and two Diploma nurse Anesthesia assistants were recruited, and 1-day training was given on how to complete data collection, and they were supervised by the investigators and supervisors during data collection.

To measure the incidence of EPH, the pulse oximetry (a small device that clips to a patient finger or to a patient toes, in case the patient has no finger) was used. Pulse oximetry has become an essential technology to detect, treat, and reduce the degree of hypoxemia in the developed world. Pulse oximetry has been endorsed by the Canadian Anesthesiologists’ Society, American Society of Anesthesiologists (ASA), World Federation of Societies of Anesthesiologists, and the WHO as a minimal monitoring standard during surgery (Nesthesia and Anesthe 2010). Reliably, it monitors the peripheral oxygen saturation of the patient noninvasively. The data collectors had been alerting the PACU nurses once the patient become hypoxemic to put on oxygen.

Thus, in this study, pulse oximetry was used to measure the presence of EPH among adult patients who have undergone different emergency surgery under general anesthesia at JMC. Consequently, clinically important EPH was defined as a pulse oximetry reading less than or equal to 92% at any time throughout the study time (starting from the time patient transferring to the stretcher up to 20 min in the PACU). A pulse oximetry reading with normal wave stay for more than 30 s was taken throughout the 20 min, every 5 min. As most hypoxemic events occur during the 15 min following patients’ admission to PACU (Quintero-cifuentes and Pérez-lópez 2018a), we enforced to observe for 20 min at PACU. Another factor that limits us to the first 20 min is we had been working under budget and time constraints.

To ensure data quality, training and adequate orientation were given to all data collectors. Then, the prepared format and questionnaire were given to data collectors. For validation of the questionnaire, a pre-test was conducted on 5% of study participants at Shenen Gibe Hospital, Jimma Zone, 2 weeks before the actual data collection time, and few modifications were made. During data collection, regular supervision and follow-up were provided by the principal investigator and the data was cross checked for completeness and consistency on daily basis. After the data collected checked for completeness, consistency, and accuracy, it was entered into Epi-data software version 4.6.0.4 using data entry template.

### Operational definitions

**Table:** 

EPH	Patients had been sorted as hypoxemic, if at any time during the observation period they were presented with SpO2 below or equal to 92% on room air for more than 30 s, and normoxaemic, if no SpO2 below 92% without oxygen therapy during the study period
Anesthesia ended time:	Time at which anesthesia ended was used to define it as nighttime and daytime
Nighttime	Anesthesia ended between 8:00 pm and 7:59 am
Daytime	Anesthesia ended between 8: am and 7:59 pm
Evening	6–9 pm
Late evening	9–midnight
Late at night	Midnight–6 am
Toward morning	3–6 am
Morning	6am–noon
Early-morning	6–9 am
Mid-morning	8–10 am
Late morning	9 am–noon
Afternoon	noon–6 pm
Early afternoon	noon–3 pm
Mid-afternoon	2–4 pm
Late-afternoon	3–6 pm
General Anesthesia	A combination of amnesia, analgesia, muscle relaxation, and ETT
Transferring time	Is a time of moving the patient from OR table up to PACU arrival
Muscular strength score 1	When patients keep a hand grip for > 15 s, while observing after letting them do so
Muscular strength 0	When the patient does not keep a hand grip or keep it for less than 15 s, while observing after letting them do so

The minimum sample size required was calculated using a single population proportion formula as follows:

$$n=\frac{N(\mathrm{Z\alpha }/2 ){2}P(1-P)}{d{2} \left(N-1\right)+(\mathrm{Z\alpha }/2 ){2}P(1-P)}$$

where.

*n* = minimum sample size required for the study.

*N* = number of the study population, the annual number of patients undergone emergency surgery during the study period, which is 2650.

*Z*_α/2_ = confidence Interval (CI) at 95% which is 1.96.

*d* = margin of error tolerated which is (5%).

*P* = 50%, assumed incidence of EPH at JMC.

By substituting to the above formula, the minimum sample size required for the study was 336.

Adding 5% of non-response rate, finally, a total of 352 study participants were enrolled in the study.

Consecutive sampling technique was used, i.e., all consecutive adult patients who have undergone emergency surgery under GA with ETT, who fulfilled inclusion criteria during the study period, were included and recruited prospectively until the sample size would meet. The data was collected during the day and nighttime, as emergency surgery takes place at any time of the day.

Statistical data analysis was done using SPSS software version 22. Descriptive statistics were done and presented using tables and figures. Pearson chi-square test was used to test the level of significance. A *p*-value of less than 0.05 with a 95%CI was considered to constitute a statistically significant difference. Bivariable and multivariable binary logistic regressions were conducted to see the existence of the association between dependent and independent variables. All variables that were found to be statistically significant (*p* value less than or equal to 0.2) on bivariable analysis were entered into a multivariable logistic regression analysis to determine the significant independent predictors of EPH in those particular groups of patients.

The analyzed, compiled, and organized data was compared and discussed. Consequently, the result was presented in tables and graphs, and finally, conclusions and recommendations were generated according to the result obtained.

Ethical clearance and approval were obtained from the institutional review board of Jimma University and were brought to the office of JMC, OR head. Then, a formal letter of permission to conduct the study was taken. Oral informed consent was also obtained from each study participant after a clear orientation of the study objective, benefit, and procedures. Confidentiality of participant’s information was kept using unique codes and medical record numbers rather than personal identification. Moreover, the data collected from each study participant were used solely for the intended purpose.

## Results

### Socio-demographic and clinical characteristics

A total of 352 eligible patients had participated in this study. All of the sampled patients responded to the interview (100% response rate). Out of 352, 59.4% were males. Around three-fourths, 262 (74.43%), of the participants’ age were less than 55. Majority of the patients, 236 (67.05%), during the study period were ASA class I patients. From 352 patients who have undergone emergency surgery, the magnitude of EPH was 149 (42.3%). Of patients greater than or equal to 55 years old, 67 (74.4%) showed the most frequent hypoxemia. The frequency of EPH was high, 22 (81.5%) among ASA III patients. Among sampled patients, subjects who had preoperative oxygen saturation less than ninety-five experienced the most frequent EPH, 68 (64.8%) (Table 1).

**Table 1:** Frequency distribution of socio-demographic and clinical characteristics with oxygen saturation status of the patient’s undergone emergency surgery under GA (*N* = 352)

Characteristics	Category	Oxygen saturation status in percent	
		Normal	Hypoxemic (EPH)
Sex	Male	117 (56.0)	92 (44.0)
	Female	86 (60.1)	57 (39.9)
Age	< 55	180 (68.7)	82 (31.3)
	≥ 55	23 (25.6)	67 (74.4)
BMI	< 18.5	39 (60.0)	26 (40.0)
	18.5–24.9	157 (59.7)	106 (40.3)
	25–29.9	7 (29.2)	17 (70.8)
ASA	ASA I	174 (73.7)	62 (26.3)
	ASA II	24 (27)	65 (73.0)
	ASA III	5 (18.5)	22 (81.5)
Preoperative oxygen saturation	≥ 95%	166 (67.2)	81 (32.8)
	< 95%	37 (35.2)	68 (64.8)

### Incidence of EPH at different times of measurement

**Fig. 1:**
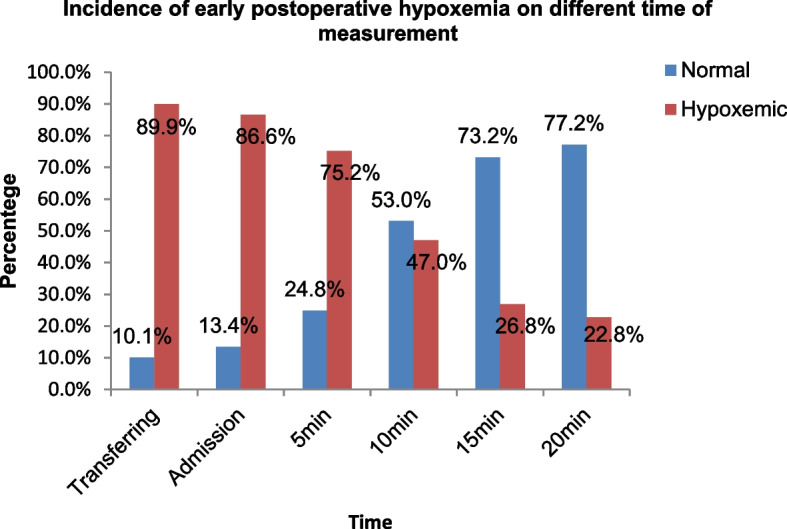
Incidence of early postoperative hypoxemia at different times of measurement

From 352 patients who have undergone emergency surgery, the magnitude of EPH was 149 (42.3%). Of 149 hypoxemic cases, EPH occurred during transferring the patients and immediately as the patients’ admission to PACU was high, 134 (89.93%) and 129 (86.58%) respectively. Low frequency, 34 (22.82%) of EPH occurred during the last 20 min. As the time increases beyond 20 min, the likelihood of EPH decreases (Fig. 1).

### Hypoxemia distribution with surgery and postoperative factors

**Fig. 2:**
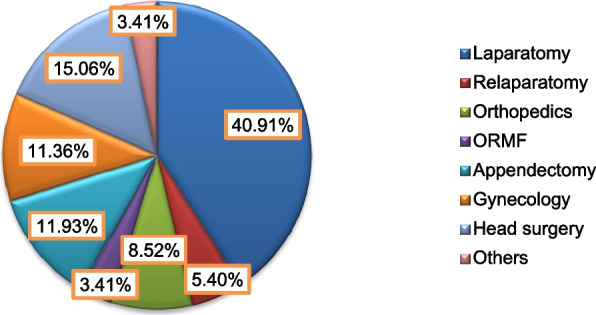
Distribution of emergecy surgery among the study subjects

As showed in Fig. 2, majority of the patients underwent emergency laparotomy and head surgery, 144 (40.91%) and 53 (15.06) respectively. The frequency of EPH was 15 (78.9%) among patients who have undergone emergency relaparotomy. But only one patient from ORMF surgery experienced EPH.

The incision site of majority of the surgeries was the upper and lower abdominal site, which accounts for 158 (44.9%). About 202 (57.4%) surgeries took greater than 2 h. More than two-thirds, 254 (72.2%), of the patients took greater than 1500-ml fluid. Around 294 (83.52%) of the patients had normal blood pressure. The frequency of EPH was high, 26 (78.8%) among patients with hypotension (Table 2). 196 patients had a muscular strength score 1, whereas 156 had a muscular strength score 0. The frequency of EPH was high (94 (60.3)) among patients with muscular strength score 0 (Table 2).

**Table 2:** Frequency distribution of surgery and postoperative factors with oxygen saturation status of patients who have undergone emergency surgery under GA (*N* = 352)

Characteristics	Category	Oxygen saturation status in percent	
		Normal	Hypoxemic
Incision site	Upper abdominal	4 (66.7)	2 (33.3)
	Lower abdominal	62 (74.7)	21 (25.3)
	Upper and lower abdominal	72 (45.6)	86 (54.4)
	Limbs	20 (66.7)	10 (33.3)
	Others (head, neck…)	45 (60)	30 (40)
Surgeon	Senior	5 (62.5)	3 (37.5)
	Resident	170 (57.4)	126 (42.6)
	Both	28 (58.3)	20 (41.7)
Surgery duration	< 120 min	107 (71.3)	43 (28.7)
	≥ 120 min	96 (47.5)	106 (52.5)
Fluid	< 1500 ml	74 (75.5)	24 (24.5)
	≥ 1500 ml	129 (50.8)	125 (49.2)
EBL	< 500 ml	184 (61.5)	115 (38.5)
	≥ 500 ml	19 (35.8)	34 (64.2)
BP	Normal	185 (62.9)	109 (37.1)
	Hypotensive	7 (21.2)	26 (78.8)
	Hypertensive	11 (44)	14 (56)
Muscular strength	Score 0	62 (39.7)	94 (60.3)
	Score 1	141 (71.9)	55 (28.1)

### EPH and time anesthesia ended

**Fig. 3:**
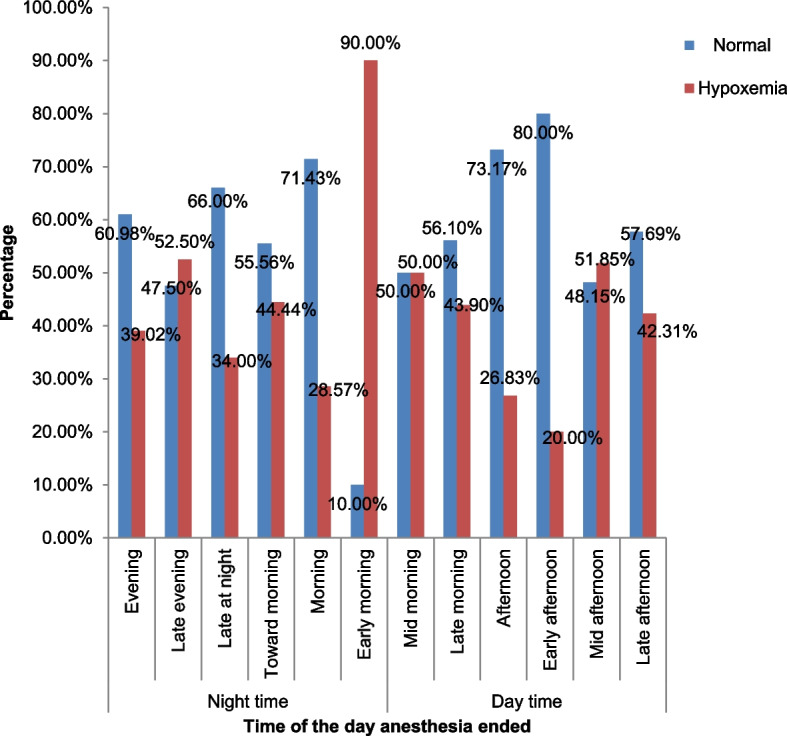
Time of the day anesthesia ended

The incidence of EPH was more frequent among patients who have undergone surgery during the nighttime 82 (46.6%) than day time, 67 (38.1%). Anesthesia ended at early morning or nighttime was accompanied by high frequency of EPH, 18 (90%) (Fig. 3).

### EPH distribution with anesthesia-related factors

The inhalational anesthetic used for the majority, 238 (67.6%), of the surgeries was isoflurane. More than three-fourths, 299 (84.94%), of the patients took both suxamethonium and vecuronium for relaxation and reversal was given for the majority of them, 314 (89.2%) (Table 3).

**Table 3:** Frequency distribution of anesthetic-related factors with oxygen saturation of patients who have undergone emergency surgery under GA at JMC from March 01 to May 30, 2021 (*N* = 352)

Characteristics	Category	Oxygen saturation status in percent	
		Normal	Hypoxemic
Inhalational anesthetics	Halothane	53 (47.3)	59 (52.7)
	Isoflurane	149 (62.6)	89 (37.4)
	Both (halothane and isoflurane)	1 (50)	1 (50)
IV anesthetic drug	Ketamine	92 (55.1)	75 (44.9)
	Thiopentone	49 (62.8)	29 (37.2)
	Propofol	40 (63.5)	23 (36.5)
	Ketofol	22 (50)	22 (50)
Muscle relaxants	Suxamethonium	24 (57.1)	18 (42.9)
	Vecuronium	5 (45.5)	6 (54.5)
	Both (sux and vecuro)	174 (58.2)	125 (41.8)
Reversal (Neostigmine)	Yes	182 (58)	132 (42)
	No	21 (55.3)	17 (44.7)
Opioids	Pethidine	180 (59.4)	123 (40.6)
	Morphine	18 (46.2)	21 (53.8)
	Tramadol	5 (50)	5 (50)

### Factors associated with EPH

On analyzing in the bivariable analysis, 13 variables had *p* value less than or equal to 0.2 and run into multivariate analysis, on which seven variables were continued to be associated with EPH (*p* value < 0.05). Six variables which were failed on analyzing with multivariate logistic regression include BMI, amount of fluid given, EBL, type of procedures, pain, and incision site. On multivariable analysis, ASA class III [(AOR, 95%CI) 12.35, (4.50–34.02)], preoperative oxygen saturation less than 95% [(AOR, 95%CI), 2.371, (1.35–4.16)], age greater than 55 years [(AOR, 95%CI), 3.163, (1.7–5.91)], nighttime anesthesia ended [(AOR, 95%CI) 1.760, (1.01–3.05)], surgery duration greater than 120 min [(AOR, 95%CI), 2.012, (1.2–3.51)], muscular strength score 0 [(AOR, 95%CI), 2.944, (1.80–4.82)], and hypotension [(AOR, 95%CI), 10.3, (2.40–44.16)] demonstrated statistically significant association with EPH (Table 4).

**Table 4:** Bivariable and multivariable regression analysis results show the independent risk factors of EPH among patients undergoing emergency surgery at JMC, 2021(*N* = 352)

Variables	Category	Frequency (%)		COR (95%CI)	AOR (95%CI)	*P* value
		Normal	Hypoxemic			
ASA (III)	Yes	5 (18.5)	22 (81.5)	2.27 (0.64, 8.1)	12.35 (4.50, 34.02)	< 0.001
	No	198 (60.9)	127 (39.1)	1	1	
Muscular strength	Score 0	62 (39.7)	94 (60.3)	1.84 (0.99, 3.42)	2.944 (1.80, 4.82)	< 0.001
	Score 1	141 (71.9)	55 (28.1)	1	1	
Age	≥ 55	23 (25.6)	67 (74.4)	3.62 (1.72, 7.61)	3.163 (1.7, 5.91)	< 0.001
	< 55	180 (68.7)	82 (31.3)	1	1	
Preoperative oxygen saturation	< 95%	37 (35.2)	68 (64.8)	2.72 (1.38, 5.35)	2.371 (1.35, 4.16)	0.003
	≥ 95%	166 (67.2)	81 (32.8)	1	1	
BP (hypotensive)	Yes	7 (21.2)	26 (78.8)	9.15 (1.79, 46.82)	10.3 (2.40, 44.16)	0.002
	No	196 (61.4)	123 (38.6)	1	1	
Surgery duration	≥ 120 min	96 (47.5)	106 (52.5)	2.53 (0.74, 8.6)	2.012 (1.2, 3.51)	0.014
	< 120 min	107 (71.3)	43 (28.7)	1	1	
Time anesthesia ended	Night	94 (53.4)	82 (46.6)	2.01 (1.1, 3.1)	1.760 (1.01, 3.05)	0.045
	Day	109 (61.9)	67 (38.1)	1	1	

## Discussion

The main finding of this study showed that the magnitude of EPH was 42.3%. This finding was high compared with previous study conducted at Gondar (26.7%) (Melesse DY, Denu ZA, Kassahun HG, Agegnehu AF. The incidence of early post-operative hypoxemia and its contributing factors among patients underwent operation under anesthesia at University of Gondar comprehensive and specialized referral hospital, Gondar, North West Ethiopia 2018). These high differences may be attributed to, due only emergency surgery, emergency surgery under general anesthesia with ETT and the cutoff point of SpO2 less than or equal to 92 were used in this study. Moreover, the current incidence was also high as compared with the study conducted in Canada (20%) (Siddiqui et al. 2006). The possible reason might be, in the previous study, less than 50% of the patients were transported with oxygen, which was not at all in the current study. A retrospective study done in China demonstrated that the incidence of EPH was 49.5% (Wang et al. 2013). However in the current study, the incidence was lower. This variation might be tantamount to the previous study subjects, who underwent aortic dissection, highly risk patients.

In the current study, ASA III had a significant association with EPH. ASA class III patients were 12 times more likely to develop EPH than their counterparts. This finding was consistent with studies in Brazil, Thailand, and Denmark (Filho et al. 2001; Moller et al. 1990; Charuluxananan 2007). This could be because those patients, ASA class III, are associated with moderate to severe systemic diseases such as respiratory system and cardiovascular system with greater risk of hypoxemia.

Moreover, the current study revealed that a muscular strength score 0 had a significant association with EPH. Patients with a muscular strength score 0 were 3 times more likely to develop EPH than patients with a muscular strength score 1. The result was in agreement with studies done in Qatar, Brazil, and Ethiopia (Faraj et al. 2012; Filho et al. 2001; Melesse DY, Denu ZA, Kassahun HG, Agegnehu AF. The incidence of early post-operative hypoxemia and its contributing factors among patients underwent operation under anesthesia at University of Gondar comprehensive and specialized referral hospital, Gondar, North West Ethiopia 2018). The amenable explanation may be due to the fact that muscular strength zero indicates there is residual neuromuscular blockade which impairs the muscles of the respiratory system (Murthy et al. 2012). As indicated in Table 3, 44.7% of patients who were hypoxemic did not get neuromuscular reversal and this leads to residual neuromuscular blockage marked by muscular strength score 0. This is of utmost importance in ensuring the necessity of neuromuscular reversal.

Likewise, the current study vividly delineated that age greater than 55 had a significant association with EPH. Patients aged over 55 years were 3 times more likely to develop EPH than patients less than 55 years. This finding goes in line with previous studies done in China, India, Brazil, and Colombia (Filho et al. 2001; Tang et al. 2019; Maity et al. 2010; Quintero-cifuentes and Pérez-lópez 2018b). The gradual decline of organ function and a residual NMB was because of altered pharmacokinetics when there is diminished liver, heart, and lung reserve (Murphy et al. 2015). However the study conducted in Gondar, Ethiopia, did not find a significant association of advanced age with EPH (Melesse DY, Denu ZA, Kassahun HG, Agegnehu AF. The incidence of early post-operative hypoxemia and its contributing factors among patients underwent operation under anesthesia at University of Gondar comprehensive and specialized referral hospital, Gondar, North West Ethiopia 2018). The possible reason might be, during the previous study regional anesthesia had been given to those patients, which put patients less likely to develop EPH. This is not an issue in the current study as it is designed only for these patients who undergo emergency surgery only under GA with ETT. Another possible explanation might be in the previous study majority of the patients were undergoing elective surgery.

In addition, this paper also found that preoperative oxygen saturation less than 95% had a significant association with EPH. Patients with preoperative oxygen saturation less than 95% were 2 times more likely to develop EPH than patients who were not. This is consolidated by prospective observational study done in India, which found that preoperative oxygen saturation less than 96% was an independent associated factor of EPH (Kaushal et al. 2018). The possible explanation underlying might be preoperative oxygen saturation less than 95% might be a surrogate of preoperative patients’ respiratory function impairment.

It is noteworthy that the study also found surgery duration greater than 2 h had a significant association with EPH. Study subjects who have undergone surgeries for greater than 2 h were 2 times more likely to develop EPH than patients who have undergone surgery for less than 2 h in this study. This is consistent with the previous study done at Victoria General Hospital in Halifax, Canada, to identify risk factors of EPH and found that longer surgery duration was one of the significant risk factors (Walker et al. 2015).

In this current body of paper, hypotension was also found as one of the significant predictors of EPH. Patients who experienced hypotension were 10 times more likely to suffer from EPH. The study done in Denmark revealed that hemodynamic instability could cause EPH (Moller et al. 1990). However different studies done on elective surgery did not identify hypotension as a significant association factor of EPH because the likelihood of hypotension in these patients is low due to optimization before surgery. But in case of emergency surgery as there is no sufficient time, optimization before surgery is rare. Patients with hypotension experience hypoxemia due to hypo-perfusion (Matteo 1980).

Excitingly, the current study found that nighttime, a factor which was not ever studied with EPH, had a significant association with EPH. Patients who have undergone surgeries during the nighttime were 2 times more likely to develop EPH than their counterparts. Even if there are no previous studies done regarding the EPH and time of the day anesthesia ended, we believe that the robust circadian rhythm of the lung is powerful enough to explain this finding. The pulmonary autonomic nerve supply has a circadian rhythmic activity. It has maximal parasympathetic activity in the early morning and maximal sympathetic activity in the late afternoon. This leads to a circadian rhythm in the bronchial tone with maximal broncho-constriction about 6:00 AM and maximal bronchodilation at about 6:00PM (Guyton and Hall 2017). The anesthesia drug effect again may superimpose it. Expecting a more advanced future study on the circadian rhythm of the lung and EPH, the current study advocates circadian rhythm of the lung favors early morning time anesthesia-ended-related risk of EPH, particularly early morning early postoperative hypoxemia (EMEPH).

### Study strengths and limitations

Exhaustively collecting data during the day and nighttime among patients who have undergone emergency surgery under GA with ETT was one of the strengths of this study. This enabled the study to find out new factor of EPH and add new knowledge to the existing body of literature. The other strength of this study is that our findings will be evidence and prompt policy maker to pay attention to the area in providing the PACU with sufficient oxygen, thereby decreasing the incidence rate of EPH. Our study had limitations too; firstly the study did not follow these patients who developed EPH for a long time to reach at secondary complications that accompany EPH. Another is that this was a single institution study with a limited sample size and included only adult patients who have undergone surgery at JMC and were admitted to PACU.

## Conclusion

The finding of this study concludes the magnitude of EPH was nearly half the study participants. Age greater than 55, muscular strength score 0, ASA III, surgery duration greater than 2 h, hypotension, and preoperative oxygen saturation less than 95% were found to be factors significantly associated with EPH. Additionally, the study found that anesthesia ended during nighttime was significantly associated with EPH, particularly EMEPH. In culmination, the study concludes there is anesthesia ended time-related risk of EPH, in addition to previously found predictors of EPH.

## Data Availability

The datasets used and/or analyzed during the current study are available from the corresponding author on reasonable request.

## Abbreviations

AOR: Adjusted odds ratio
EMEPH: Early morning early postoperative hypoxemia
EPH: Early postoperative hypoxemia
ETT: Endo-tracheal tube
GA: General anesthesia
JMC: Jimma Medical Center
OR: Operation room
PACU: Postoperative care unit
PaO2: Arterial partial pressure of oxygen
SpO2: Pulse oximetry that measures oxygen saturation peripherally
